# Effect of Solution Conditions on the Properties of Sol–Gel Derived Potassium Sodium Niobate Thin Films on Platinized Sapphire Substrates

**DOI:** 10.3390/nano9111600

**Published:** 2019-11-11

**Authors:** Alexander Tkach, André Santos, Sebastian Zlotnik, Ricardo Serrazina, Olena Okhay, Igor Bdikin, Maria Elisabete Costa, Paula M. Vilarinho

**Affiliations:** 1 Department of Materials and Ceramic Engineering, CICECO–Aveiro Institute of Materials, University of Aveiro, 3810-193 Aveiro, Portugal; 2 Łukasiewicz Research Network-Institute of Electronic Materials Technology (ITME), Wolczynska 133, 01-919 Warsaw, Poland; 3 Nanotechnology Research Division, Centre for Mechanical Technology and Automation (TEMA), Department of Mechanical Engineering, University of Aveiro, 3810-193 Aveiro, Portugal

**Keywords:** local piezoresponse, dielectric properties, K_0.5_Na_0.5_NbO_3_ (KNN) thin film morphology and texturing, solution stoichiometry and concentration

## Abstract

If piezoelectric micro-devices based on K_0.5_Na_0.5_NbO_3_ (KNN) thin films are to achieve commercialization, it is critical to optimize the films’ performance using low-cost scalable processing conditions. Here, sol–gel derived KNN thin films are deposited using 0.2 and 0.4 M precursor solutions with 5% solely potassium excess and 20% alkali (both potassium and sodium) excess on platinized sapphire substrates with reduced thermal expansion mismatch in relation to KNN. Being then rapid thermal annealed at 750 °C for 5 min, the films revealed an identical thickness of ~340 nm but different properties. An average grain size of ~100 nm and nearly stoichiometric KNN films are obtained when using 5% potassium excess solution, while 20% alkali excess solutions give the grain size of 500–600 nm and (Na + K)/Nb ratio of 1.07–1.08 in the prepared films. Moreover, the 5% potassium excess solution films have a perovskite structure without clear preferential orientation, whereas a (100) texture appears for 20% alkali excess solutions, being particularly strong for the 0.4 M solution concentration. As a result of the grain size and (100) texturing competition, the highest room-temperature dielectric permittivity and lowest dissipation factor measured in the parallel-plate-capacitor geometry were obtained for KNN films using 0.2 M precursor solutions with 20% alkali excess. These films were also shown to possess more quadratic-like and less coercive local piezoelectric loops, compared to those from 5% potassium excess solution. Furthermore, KNN films with large (100)-textured grains prepared from 0.4 M precursor solution with 20% alkali excess were found to possess superior local piezoresponse attributed to multiscale domain microstructures.

## 1. Introduction

Owing to the environmental issues with the state-of-the-art piezoelectric materials based on lead zirconate titanate and other lead containing compounds, potassium sodium niobate (K_0.5_Na_0.5_NbO_3_, KNN) has become an extensively investigated system [1] due to its elevated Currie temperature (*T_C_*), up to 420 °C, and high longitudinal piezoelectric coefficient (*d*_33_) reported by Saito et al. on KNN-based ceramics in 2004 [2]. Since that time, an increasing interest has been focused on KNN-based bulk materials [1,2,3,4] as possible alternatives in various applications, such as sensors, actuators, energy harvesting and microelectromechanical systems (MEMS) [5]. Considering that, numerous efforts have been made by researchers to fabricate KNN and KNN-based thin films due to their potential applications in sensor and actuator micro-devices, as well as miniaturized MEMS [6]. However, the reported piezoelectric performance for KNN-based thin films is significantly inferior compared to that of bulk KNN-based materials mainly due to the volatility of alkaline elements, composition deviation from stoichiometry, issues related to the formation of secondary phases, and high leakage current density [6].

Certain advances in the electrical properties of KNN-based thin films have been observed upon the addition of K and/or Na excess in the precursors of the sol–gel derived KNN thin films [6,7,8]. In particular, Ahn et al. reported that when the solution contained 20% alkali-excess, the preferentially (100) oriented films revealed the best electrical properties [7]. The 250 nm thick films were prepared from solutions with 0.1 M concentration, performing final heating by insertion into furnace at 700 °C for 30 min. Further, Kupec et al. have shown that the optimal electrical response can be observed at only 5% excess of potassium, when the 250 nm thick films are prepared from a solution with 0.4 M concentration and annealed at 750 °C for 5 min using rapid thermal annealing (RTA) [8].

However, there has been no systematic study presenting solely the solution concentration effect on the properties of KNN thin films. Moreover, although such studies have been reported for 1 µm thick Pb(Zr_0.52_Ti_0.48_)O_3_ (PZT) films on Pt(111)/Ti/SiO_2_/Si(100) [9] and 400 nm thick 0.755Bi_0.5_Na_0.5_TiO_3_–0.065BaTiO_3_–0.18SrTiO_3_ (BNT–BT–ST) films on LaNiO_3_(100) buffered Pt(111)/Ti/SiO_2_/Si substrates [10], their results are rather contradictive. The PZT films were found to be more (111) textured with increasing solution concentration that resulted in an enhanced piezoresponse [9]. In contrast, higher piezoresponse was reported for (100) oriented BNT–BT–ST films obtained from 0.1 M solution compared to that for randomly oriented films prepared from the solution with a higher concentration of 0.2 M [10]. Therefore, each material can have its own solution concentration—texturing—piezoresponse relationship that has to be established.

Furthermore, in our recent work we have shown that substrates, used for the film deposition, also have a determining role on the final properties of the films [11]. Thus, platinized Si, mainly reported as substrate for KNN [6,7,8] and other piezoelectric films [9,10], has been shown to induce large tensile residual stress in sol–gel derived undoped KNN films due to thermal expansion mismatch, thus diminishing out-of-plane dielectric, ferroelectric, and piezoelectric response of the films [11]. On the other hand, platinized SrTiO_3_ substrates, possessing much larger thermal expansion coefficient (TEC), have been shown to induce residual compressive stress in KNN films, significantly enhancing out-of-plane permittivity, polarization, and piezoresponse [11]. Therefore, to study only the influence of the solution conditions on the properties of polycrystalline KNN thin films, minimizing the effect of the substrate, one should choose a substrate with similar TEC. In the case of KNN, sapphire (Al_2_O_3_) has very similar thermal expansion properties. Nevertheless, sol–gel derived KNN thin films deposited on platinized sapphire have not yet been studied. To the best of our knowledge, there was just a report on over 10 µm thick films of KNN with complex perovskite materials, aerosol deposited on platinized sapphire [12], and a recent article on 510 nm thick KNN films, pulsed laser deposited on sapphire without a Pt layer [13].

In this work and within this context, crack-free KNN thin films are prepared by chemical solution deposition from 0.2 and 0.4 M precursor solution with 5% solely potassium excess and 20% alkali (potassium and sodium) excess on platinized sapphire substrates, being then annealed using RTA. Performing atomic force microscopy (AFM), scanning electron microscopy (SEM) and energy dispersive spectroscopy (EDS) analyses in combination with X-ray diffraction, dielectric spectroscopy and piezo-force microscopy (PFM) characterization, a correlation between the electrical properties in the parallel plate capacitor geometry, piezoresponse and the solution conditions for KNN films deposited on platinized Al_2_O_3_ was found.

## 2. Experimental Details

### 2.1. Fabrication

For the synthesis of KNN films, a precursor solution was prepared using potassium acetate (≥99%, ChemPur GmbH, Karlsruhe, Germany), sodium acetate (99%, Alfa Aeasar, Haverhill, MA, USA), niobium pentaethoxide (99%, H.C. Starck Tantalum and Niobium GmbH, Goslar, Germany), and 2-methoxyethanol (99%, Sigma Aldrich, Saint Louis, MO, USA). Initially, 2-methoxyethanol was placed in a closed flask and left for 30 min in N_2_ under constant stirring. Using a glove box with Ar atmosphere, potassium acetate, sodium acetate, and niobium pentaethoxide were weighted according to the requested ratio and dissolved in 2-methoxyethanol. A 5 mol % excess of potassium (corresponding to composition K_0.525_Na_0.5_NbO_3_) and 20 mol % excess of potassium and sodium (corresponding to composition K_0.6_Na_0.6_NbO_3_) were used in this study to compensate for alkali volatilization during the heat treatment following works by Kupec et al. [8] and by Ahn et al. [7], respectively. After mixing the reagents, the solutions remained under stirring in N_2_ for ~30 min. Then, they were refluxed for 4 h, and distilled at 124 °C. After cooling down, the KNN precursor solutions were transferred to a flask, and 2-methoxyethanol was added to keep the concentration at 0.2 M. In the case of precursor solution with 20% excess of potassium and sodium, a part of the solution was also prepared with concentration of 0.4 M. Thus, three kinds of the KNN solutions listed in Table 1 and marked as 5%_0.2 M, 20%_0.2 M and 20%_0.4 M were prepared and used for the film deposition. The precursor KNN solutions, passed through a 0.2 µm filter, were spin-coated (Chemat Technology spin-coater KW-4A, Los Angeles, CA, USA) on platinized Al_2_O_3_ substrates at 3000 rpm for 30 s, forming layered films. For 0.2 M solutions the number of layers was 10, whereas for 0.4 M solution only 5 layers were deposited to get the films with close thicknesses. (0001)-oriented Al_2_O_3_ substrates with lateral TEC of 5.4 × 10^−6^ K^−1^ that is very close to 4.72 × 10^−6^ K^−1^ reported for KNN [14] were purchased from Crystal GmbH, Berlin, Germany, with subsequent platinization in Inostek Inc, Ansan, Korea. Before KNN deposition, the substrates were cleaned in boiling ethanol, and dried on a hot-plate. Pyrolysis of each as-deposited layer was performed at 350 °C for 2 min on a hot-plate in air. After the multicycle deposition, KNN films were annealed in air at 750 °C for 5 min with a heating/cooling rate of 30 °C/s, using an RTA system (Qualiflow, Jipelec, JetFirst 200, Montpellier, France).

### 2.2. Characterization

The thin film crystal phase evolution was analyzed by XRD with a X’Pert MPD/MRD X-ray diffractometer (Philips, Amsterdam, Netherlands), equipped with a mobile arm, using Cu Kα radiation. The θ–2θ scan technique was adopted to collect the diffraction intensity data from 20° to 60° with a 0.026° step mode. The X-ray pole figure measurements were performed using (100) reflection of KNN at about 22.1°. The surface and cross-sectional morphologies, as well as the thickness and elemental maps of KNN thin films, were obtained by scanning electron microscopy (SEM) using a field emission SEM (Hitachi, SU-70, Tokyo, Japan). Compositional analysis of the films was done by built-in energy dispersive spectroscopy (EDS) system (Bruker, QUANTAX 400, Billerica, MA, USA) in the cross-section geometry under the accelerating voltage of 8 kV, to reduce the contribution from the substrate. Dielectric and ferroelectric measurements were performed at room temperature using Au, sputtered through a mask onto the films, as top electrode, and the substrate Pt layer as the bottom one. ε_r_ and tan δ were obtained by impedance spectroscopy measurements under an applied voltage oscillation level of 50 mV in a frequency range of 10^2^–10^6^ Hz, using a precision LCR meter (Agilent, E4980A, Santa Clara, CA, USA). For the film roughness determination and piezoelectric force microscopy (PFM) characterization, a modified commercial atomic force microscope (Veeco AFM Multimode Nanoscope (IV) MMAFM-2, Santa Barbara, CA, USA) with conductive Pt-coated Si tip cantilevers, and an external lock-in amplifier (EG&G 5205, Princeton, NJ, USA) was employed. The topography images were processed using WSxMbeta6_0 software (Nanotec, Feldkirchen, Germany).

## 3. Results and Discussion

**Figure 1:**
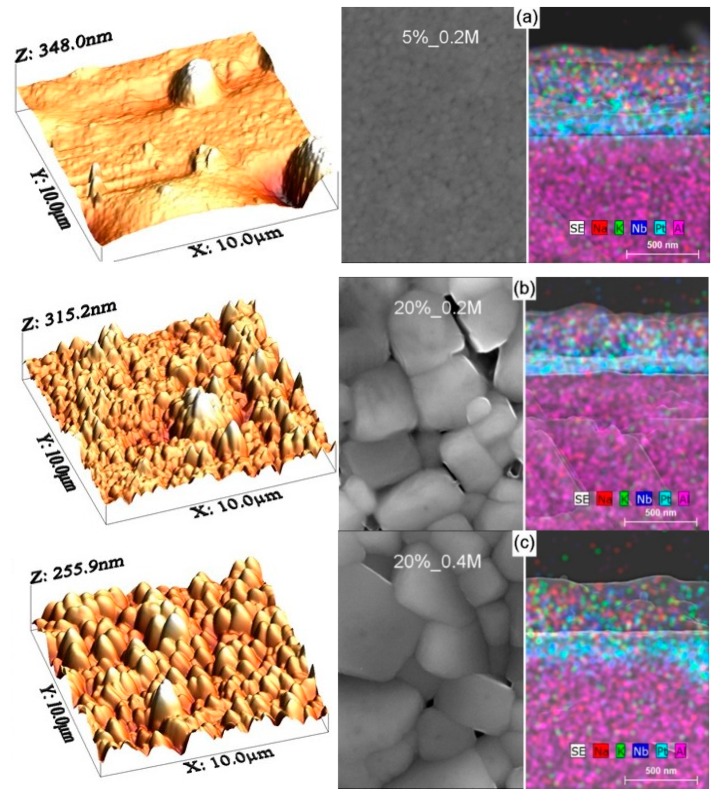
AFM (left), SEM top-view (centre) and SEM cross-section (with elemental map) micrographs (right) of KNN thin films deposited on platinized Al_2_O_3_ substrates from solutions with 5% potassium excess and 0.2 M concentration (**a**), 20% alkali excess and 0.2 M concentration (**b**) and 20% alkali excess and 0.4 M concentration (**c**). Note that the scale bar is the same for both top and cross-section views.

Figure 1a–c shows the typical AFM surface morphology as well as SEM top-view and cross-sectional microstructures of KNN thin films deposited on platinized Al_2_O_3_ substrates from three solutions under study. The surface roughness, obtained from the AFM data, as well as the average lateral grain size and the film thickness, deduced from the field emission SEM analysis, are presented in Table 1. In particular, the Table 1 shows that root mean square (RMS) values of surface roughness in a 10 µm × 10 µm area of 37 ± 2 nm for all three kinds of the KNN films under study. In the case of 5%_0.2 M films, however, the roughness in a 2 µm × 2 µm area is as low as 3 nm, but some holes and hills of µm size result in the average roughness of 39 nm for the 10 µm × 10 µm area.

No peelings or cracks were found at the dense film surfaces both at AFM (left part of Figure 1) and SEM top-view images (central part of Figure 1). At the same time, there is an evident lateral grain size variation from ~110 nm in average for 5%_0.2 M films to ~480 and ~600 nm for 20%_0.2 M and 20%_0.4 M films, respectively, as also indicated in Table 1. Thus, besides a strong increase of the grain size with the alkali excess amount observed previously [7,8] there is also slight grain size increase with the solution concentration.

The right part of Figure 1 shows simultaneously the SEM cross-sectional microstructure and EDS elemental maps of the KNN thin films grown on platinized Al_2_O_3_ substrates using three different alkali excess and/or concentration precursor solutions (for separated elemental maps and SEM cross-sectional microstructure see Figures S1–S3 in Supplementary Materials). As expected, the average film thickness of 340 ± 10 nm is similar for all three kinds of the films, whereas elemental maps give a possibility for clear determination of the KNN films consisting of Na, K, and Nb oxides, the Pt electrode layer and the Al oxide substrate. Moreover, for 5%_0.2 M films, there are several grains across the film thickness, whereas for 20%_0.2 M and 20%_0.4 M films, some regions with single grains across the film thickness are seen in agreement with the lateral grain size variation.

**Figure 2:**
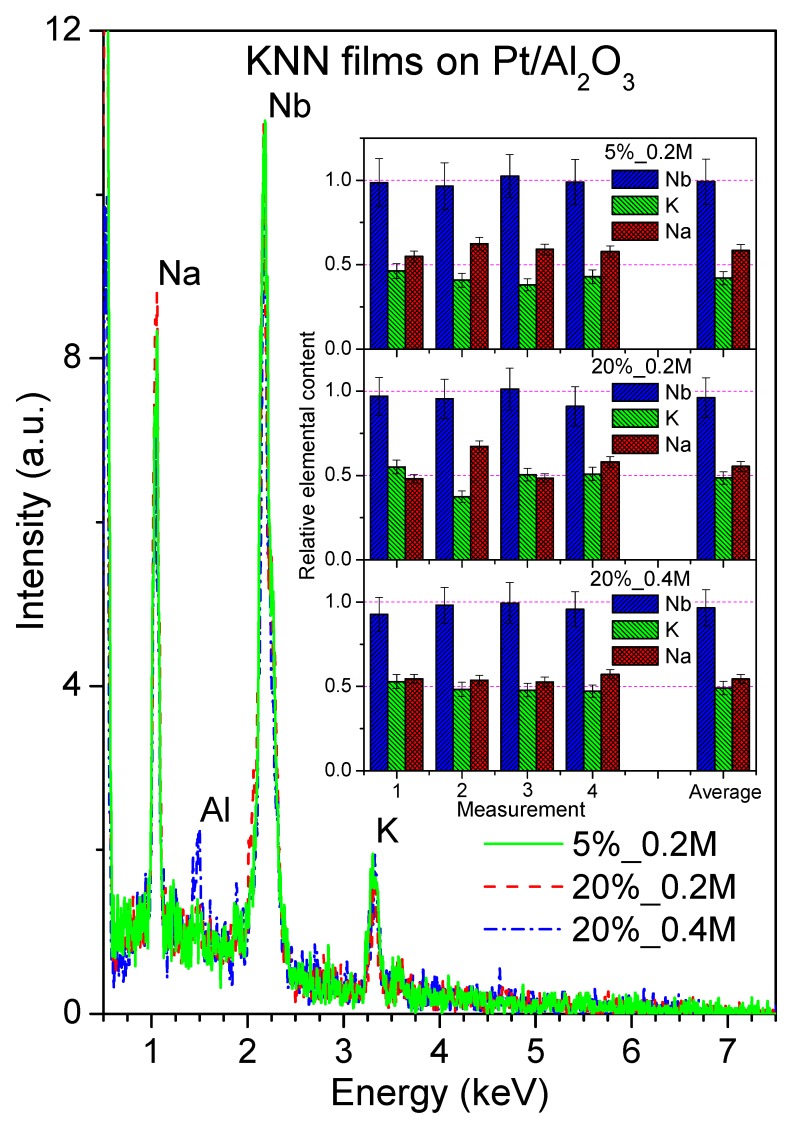
Energy-dispersive spectra of KNN films deposited on platinized Al_2_O_3_ from solutions with 5% potassium excess and 0.2 M concentration, 20% alkali excess and 0.2 M concentration, and 20% alkali excess and 0.4 M concentration. Inset shows relative elemental content of Nb, K, and Na obtained by the spectra analysis in the 4 representative spots and in average.

Further, the EDS analysis was performed on KNN thin films to estimate their chemical composition. Typical energy dispersive spectra are depicted in Figure 2, clearly presenting Na, Nb and K peaks. In the case of 20%_0.4 M films, there is also a separated peak of Al, possibly from the sample holder. The relative elemental content of K, Na, and Nb in the 4 representative spots and in average for the investigated KNN films are plotted in inset of Figure 2, and the derived (K + Na)/Nb ratio is presented in Table 1.

The results of the EDS analysis of all the KNN films reveal that the contents of K, Na, and Nb deviate for a few atomic percent from the respective stoichiometric values, which in relation to Nb content are 50 at % of K, 50 at % of Na, and 100 at % of Nb. In average, the films demonstrate some excess of alkali content in relation to that of Nb (shown also in Table 1) and a slightly higher content of Na compared to K with regard to the stoichiometric KNN composition values. The average values of K and Na fraction in relation to that of Nb in 5%_0.2M films are 42 ± 4 and 59 ± 3 at %, respectively. These values are well comparable to those of 45 ± 6 and 56 ± 6 at %, obtained for K and Na, respectively, in KNN films deposited on platinized Si substrates from solutions with 5% excess of potassium and 0.4 M concentration by Kupec et al. [8]. However, since in both our and cited cases, the films were prepared from K-rich solutions, while Na content cannot be higher than 50 at %, the observed Na fraction > 50 at % and thereby Na/K ratio > 1 cannot be explained other than by some overestimation of Na and/or underestimation of K content during the EDS fitting treatment. Despite that, we can still do some comparison between the films under study.

Thus, the films with 20% of alkali excess contain in average a larger fraction of K but pretty much the same fraction of Na, compared to 5%_0.2M films, that results in an increase of the (Na + K)/Nb ratio from 1.01 to 1.08 in the case of 20%_0.2M and to 1.07 in the case of 20%_0.4M films. Namely, 20%_0.2 M films possess 50 ± 4 at % of K and 58 ± 3 at % of Na, while the average values for K and Na in 20%_0.4 M films are equal to 51 ± 4 at % and 56 ± 3 at %, respectively. Therefore, from the absolute values, one could conclude that in both these films the most volatile element is K, of which content in the films is lower. However, taking into account that compared to the EDS analysis of the 5%_0.2 M films the excess of Na gives no increment in the Na content within the 20%_0.2M and 20%_0.4M films in contrast to K excess, the most volatile element is evidently Na. It is interesting to note that the (K + Na)/Nb ratio of 1.07 was also obtained by Kupec et al. [8] in KNN films with 10 mol % K- or 10 mol % Na-initial-excess, although such excess should not give the ratio value above 1.05. Overall, KNN films prepared by us from the solutions with 5% K excess revealed a decrease of the (K + Na)/Nb ratio from an initial 1.025 in the solution to 1.01 in the films. An alkali excess as high as 20 mol % of K and Na exhibited larger alkali losses, diminishing the ratio from 1.2 in the solution to 1.07–1.08 in the films.

**Figure 3:**
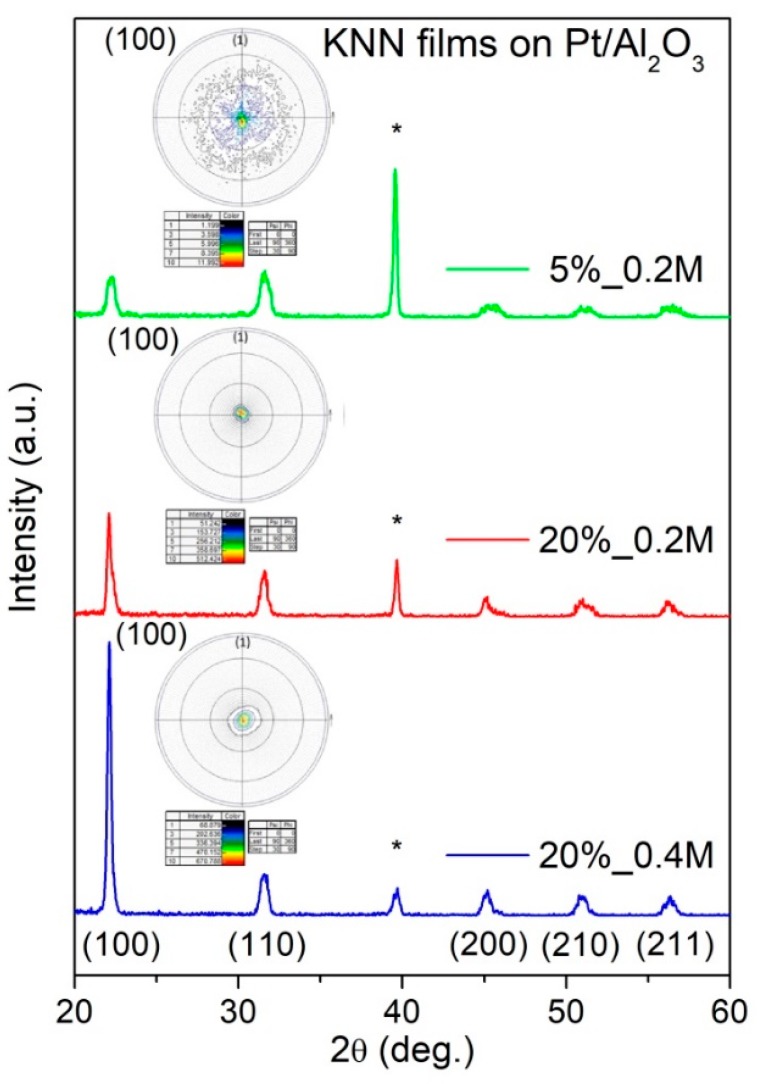
XRD patterns of KNN films deposited on platinized Al_2_O_3_ from solutions with 5% potassium excess and 0.2 M concentration, 20% alkali excess and 0.2 M concentration, and 20% alkali excess and 0.4 M concentration. Substrate peaks are marked by *. Insets show corresponding pole figures of the films measured for the (100) plane.

The increased alkali to niobium ratio could not, however, be identified by XRD, as seen from Figure 3, showing X-ray diffraction patterns of KNN thin films, prepared from the solutions with different amounts of alkali excess and/or concentrations. Besides the peak of the substrate Pt layer, only the perovskite phase peaks could be observed. Therefore, the alkali excess incorporates into the films either in the form of the amorphous alkali-rich phase or structural defects like the Ruddlesden–Popper (RP) structure in Sr-rich SrTiO_3_ [15].

The degree of the preferred orientation in the thin film samples was quantitated by the Lotgering factor (F), which ranges from 0 for a randomly oriented phase to 1 for a perfectly oriented phase [16]. The Lotgering factor for (100) preferential orientation *F*_(100)_ was calculated as

(1) $$F_{\left(100\right)}=\frac{P_{\left(100\right)}-P_{0}}{1-P_{0}}$$

where $P_{\left(100\right)}=\sum^{\;}I_{\left(100\right)}/\sum^{\;}I_{\left(hkl\right)}$ and $P_{0}=\sum^{\;}I_{\left(100\right)}^{0}/\sum^{\;}I_{\left(hkl\right)}^{0},$ with $I_{\left(hkl\right)}$ and $I_{\left(hkl\right)}^{0}$ being the intensities of (*hkl*) peaks for the textured and randomly oriented KNN thin films, respectively. Table 1 shows that there is no clear preferred orientation in the 5%_0.2 M films with an *F*_(100)_ value of just 0.13. (100) preferential orientation appears, however, with the increasing amount of alkali excess in the solution, when *F*_(100)_ value increases to 0.39 for 20%_0.2 M films. Moreover, when the solution concentration is doubled there is even higher growth of *F*_(100)_ to 0.72 for 20%_0.4 M films. Such high value indicates a significant texturing of these films along (100) crystallographic direction. The observed (100) preferential orientation for the films with high alkali excess is in agreement with other reports on alkali niobate thin films [6,7,8], although its increase with the solution concentration was not reported so far.

To have more complete characterization of the texture quality of KNN films deposited on platinized Al_2_O_3_ substrates, X-ray pole figure measurements, which reflect the preferred orientation, of the crystal lattice in the material, were performed at the fixed 2θ angle of (100) peak, as shown in the inserted illustrations of Figure 3. For the KNN thin film with 20% alkali excess, the projected intensities are all grouped in a central circle near a tilt angle of 0°. It is an obvious indication that these films are oriented along (100), as XRD patterns suggested. In contrast, KNN thin films with 5% potassium excess and 0.2 M concentration, pole figures show the most diffuse distribution of the lines indicating absence of the preferred crystallographic orientation along (100).

**Figure 4:**
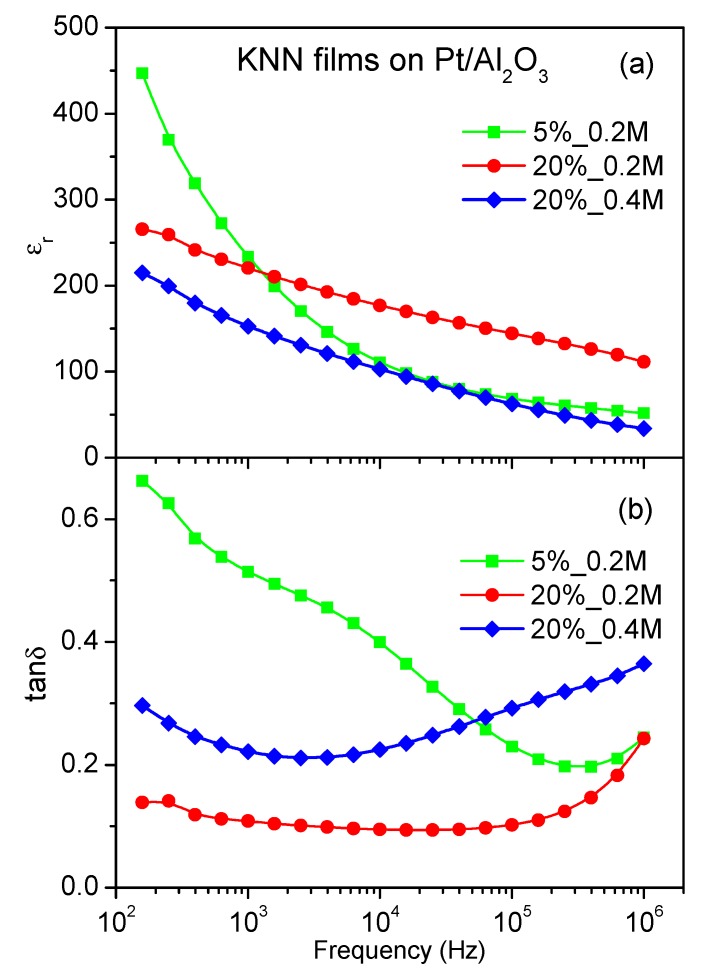
Room temperature relative permittivity ε_r_ ((**a**), solid symbols) and dissipation factor tanδ ((**b**), open symbols) as a function of frequency for KNN thin films deposited on platinized Al_2_O_3_ from solutions with 5% potassium excess and 0.2 M concentration (squares), with 20% alkali excess and 0.2 M concentration (circles), and with 20% alkali excess and 0.4 M concentration (rhombs).

The dielectric properties of the 5%_0.2 M, 20%_0.2 M, and 20%_0.4 M films grown on platinized Al_2_O_3_ and measured at room temperature as a function of frequency (10^2^–10^6^ Hz) are shown in Figure 4. All the films show similar behavior of the relative dielectric permittivity ε_r_ (Figure 4a), that is, a decrease when the frequency increases. However, for 5%_0.2 M films, the decrease is much stronger than for 20 mol % alkali excess films. Such high permittivity variation looks to be supported by two dielectric relaxations, which can be determined in the dissipation factor tanδ variation with frequency (Figure 4b).

The relative permittivity and dissipation factor values of the films at 10 kHz are listed in Table 1. These results show the highest ε_r_ and lowest tanδ for 20%_0.2 M films. Both decrease of the alkali excess content and increase of the solution concentration suppress the permittivity and enhance the dissipation factor. Films prepared from solution with just 5% excess of potassium show lower relative permittivity values at 10 kHz and stronger dielectric relaxation, comparing to that of 20%_0.2 M, due to significantly smaller grain size and possible Maxwell–Wagner-type polarization mechanism observed in the low frequency range at room temperature, respectively. In the case of the solution concentration variation, the observed variation of the dielectric response can be explained in terms of a higher (100) orientation degree of 20%_0.4 M thin films with respect to 20%_0.2 M. Indeed, some decrease of the dielectric permittivity and increase of the dissipation factor was reported for (100)-oriented KNN-based films in comparison to (110)- and (111)-oriented ones by Chen et al. [17]. Therefore, these results, although not optimized so far, indicate that there is a competition between the grain size and (100) orientation effects on the dielectric response of KNN thin films. In addition, the permittivity values of our films at 10 kHz are lower than those of 300–600 observed by Kupec et al. [8], perhaps due to higher porosity of our films. However, the reduced permittivity is not a drawback but an advantage of the materials for piezoelectric applications, if their piezoelectric properties are not suppressed [18].

**Figure 5:**
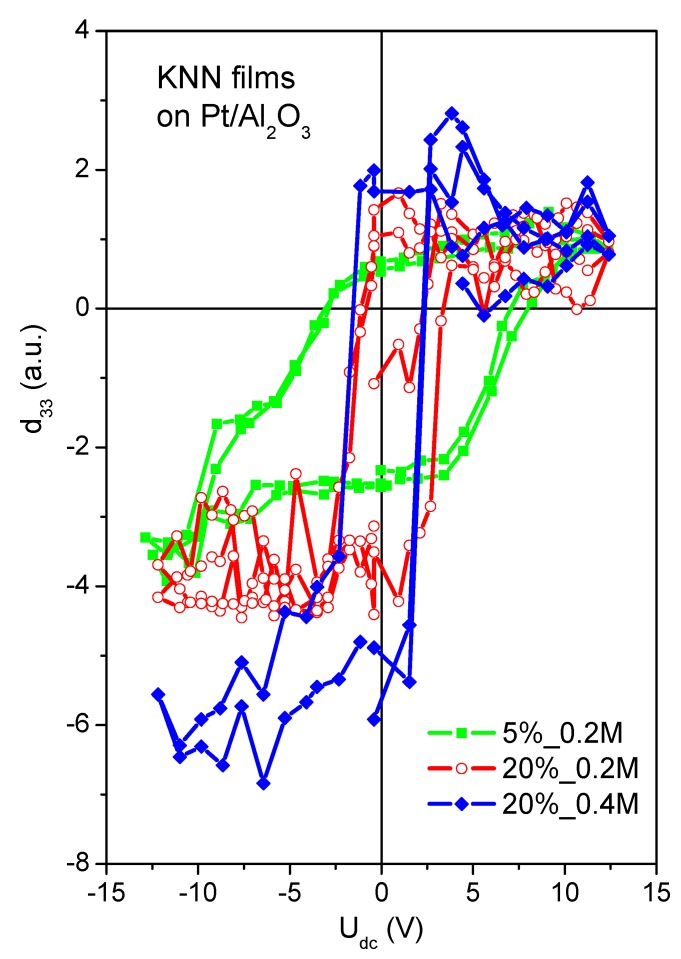
Local piezoresponse hysteresis loops of KNN thin films deposited on platinized Al_2_O_3_ from solutions with 5% potassium excess and 0.2 M concentration (squares), with 20% alkali excess and 0.2 M concentration (circles), and with 20% alkali excess and 0.4 M concentration (rhombs).

The piezoelectric properties of the KNN films estimated using PFM are presented in Figure 5, giving clear qualitative comparison for the films with similar thickness and measured at the same conditions. All the curves exhibit typical hysteresis loops, indicating the presence of piezoelectricity at least at local scale. However, the shape of the loops is more diffused in the case of 5%_0.2 M films, whereas for 20%_0.2 M and 20%_0.4 M films it is more quadratic-like. Despite of some offset, the coercive field can be estimated as about 3 times lower in the case of 20%_0.2 M and 20%_0.4 M films comparing to 5%_0.2 M films. Regarding the piezoelectric coefficient, it continuously increases from 5%_0.2 M to 20%_0.2 M and further to 20%_0.4 M films, being thus in correlation with *F*_(100)_ variation. An increase of the local piezoresponse with (100) orientation was also reported by Chen et al. [17].

It is known that the ferroelectric and electromechanical response of a material is directly related to its polar domain structure. In ferroelectric KNN thin films, energy minimization results in multiple domains, separated by domains walls. These domain walls have well-defined orientations that minimize energy by maintaining compatibility of strain and polarization across the wall. The ferroelectric domains could adopt 60°, 90°, 120°, and 180° domain walls, as in the case of KNbO_3_ and KNN [19,20]. Thus, the domain structures dictate the effective properties of the crystals.

**Figure 6:**
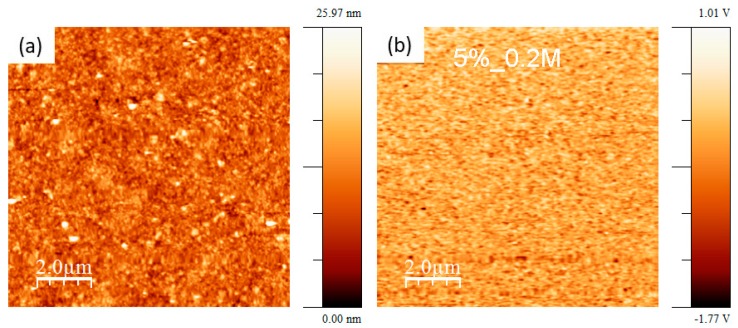
Topography (**a**) and out-of-plane piezo-force response image (**b**) of KNN thin films with 5% potassium excess and 0.2 M concentration deposited on platinized Al_2_O_3_.

Figure 6 shows topography and out-of-plane PFM signal taken on KNN thin films deposited on platinized Al_2_O_3_ substrates from solutions with 5% potassium excess and 0.2 M concentration. As the topography (Figure 6a) presents very small grains, domains with out-of-plane component of polarization are also small but numerous, as seen in Figure 6b. The dark regions in the piezo-response images represent the domains with polarization oriented towards substrate, and bright regions to domains with polarization oriented to the film surface.

**Figure 7:**
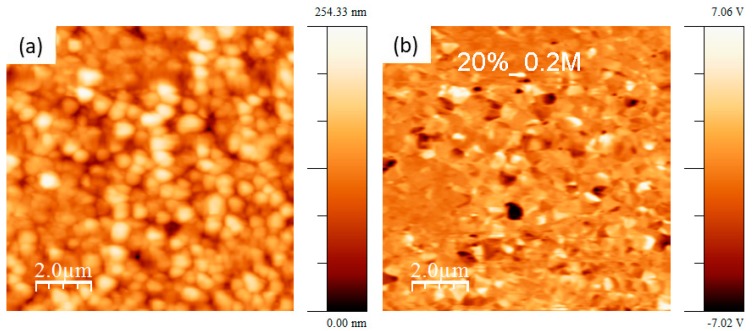
Topography (**a**) and out-of-plane piezo-force response image (**b**) of KNN thin films with 20% alkali excess and 0.2 M concentration deposited on platinized Al_2_O_3_.

Figure 7 shows topography and the out-of-plane PFM signal taken on KNN thin film deposited on platinized Al_2_O_3_ substrates using solution with 20% alkali excess and 0.2 M concentration. In this case, the contrast and domain size are higher than in the case of 5%_0.2M films, while domain number is lower.

**Figure 8:**
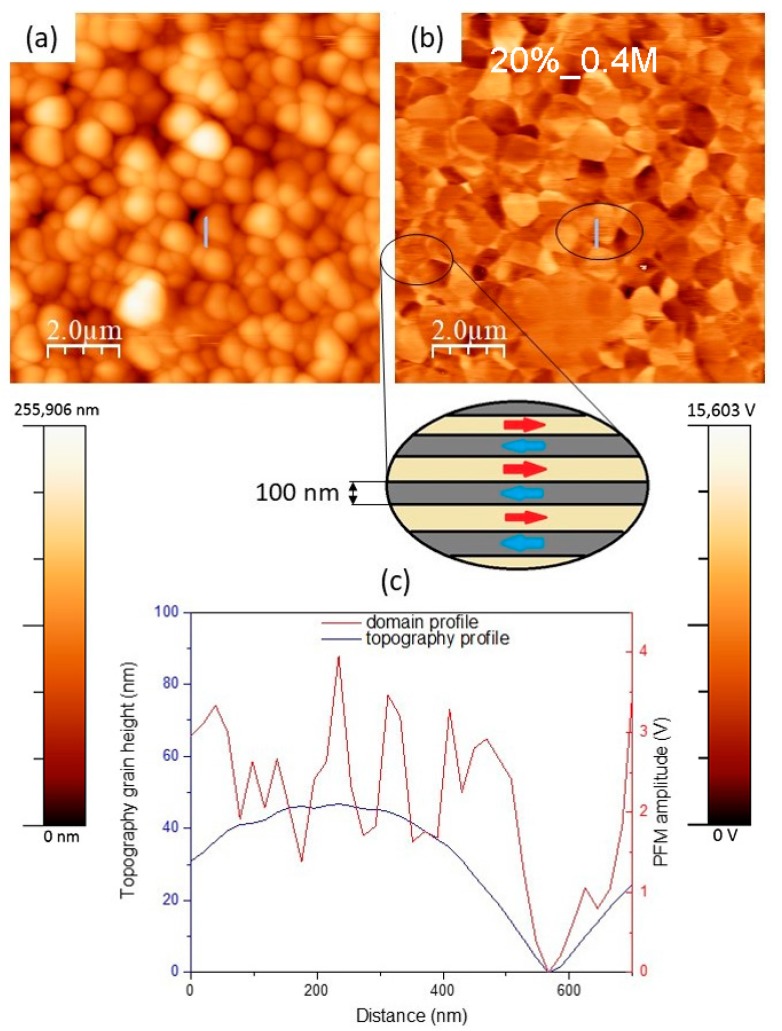
Topography (**a**), out of plane piezo-force response image (**b**), and cross-sections of topography and piezoresponse, taken along the marked line (**c**), for KNN thin films with 20% alkali excess and 0.4 M concentration deposited on platinized Al_2_O_3_. A scheme of the nanodomains observed in the circled arias is also presented.

Figure 8 shows the topography, piezo-response image, and simultaneous cross-sections of topography and PFM, taken on KNN thin films deposited on platinized Al_2_O_3_ substrate from solutions with 20% alkali excess and 0.4 M concentration. These films exhibit as some well-defined relatively large domains as nanodomains. The latter can be responsible for the enhanced piezoresponse [17]. These nanodomains are circled in Figure 8b, while other similar features corresponding to the horizontal lines in the topography (Figure 8a) should be artefacts of the PFM. The domains are separated by exactly 180° domain walls, as shown in a scheme also presented in Figure 8, with a wavy shape, characteristic to this type of the domain walls [19]. The domain length in the PFM image ranges between 500 and 750 nm and the average domain width is about 100 nm, as can be seen from the domain profile not corresponding to that of topography, as shown in Figure 8c. The observation of a 180° nanodomain structure in these films should be related with their high (100) texture and submicron grain size. Thus, very regular 180° domain structures was observed in (100) KNN single crystals, comparing to that of polycrystalline KNN [20], although the domain size was much larger than in our thin films likely due the larger crystallite size.

Although we aware that local effective piezoelectric coefficient values measured in different PFM experiments cannot be compared directly as they are highly correlated with the tip-sample contact stiffness [21] and therefore many authors of the PFM studies on KNN-based films did not even report the d*_33_ values [17,22,23], we had to estimate these values from our PFM data at least for rough comparison with those reported for KNN and other piezoelectric materials. For that, we used identical PFM measurements of a PZT thin film with known d_33_ of 160 pm/V, as a reference, and obtained values of 21, 95, and 124 pm/V for 5%_0.2 M, 20%_0.2 M, and 20%_0.4 M KNN films, respectively. The former value is just slightly larger than that of 17 pm/V for pristine KNN film [24], whereas two latter values are lower than that of 160 pm/V (pC/N) for KNN single crystals [20] but comparable with or even higher than that of 110 pm/V (pC/N) for conventionally sintered KNN ceramics [25]. Moreover, they are significantly higher than the values of 40–74 pm/V reported for other KNN-based films [24,26,27] and are comparable with the values of 110–140 pm/V reported for BNT–BT–ST films [10]. However, in contrast to the BNT–BT–ST films [10], the higher piezoresponse and (100) preferential orientation were observed in our KNN films obtained from solutions with higher concentration. Such relationship is more similar to that of PZT films, reported to possess d_33_ increasing from ~100 to ~160 pC/m with increasing solution concentration and texturing [9]. However, in that case, the texturing was along (111) but not (100) direction.

Thus, we have shown that 20%_0.4 M KNN films with the more promising piezoresponse and reduced ε_r_, compared to the films from solutions with lower concentration and/or alkali excess. These films derived by low-cost sol–gel method and deposited on well thermally matching platinized sapphire substrates, can be considered for piezoelectric applications.

## 4. Conclusions

The influence of alkali excess and concentration of solution used for deposition of sol–gel derived KNN films is studied in this work. For this purpose, polycrystalline crack-free KNN films with thickness of ~340 nm were produced from 0.2 and 0.4 M precursor solutions with 5% potassium and 20% alkali excess by RTA at 750 °C on platinized sapphire substrates. Despite of the miserable thermal expansion mismatch between the Al_2_O_3_ and KNN, it is the first report on the preparation and characterization of KNN thin films on platinized sapphire. EDS and SEM analysis has shown that nearly stoichiometric KNN films and ~100 nm average grain size were obtained using 5% potassium excess solution, while 20% alkali excess solutions gave (Na + K)/Nb ratio in the prepared films of 1.07–1.08 and the grain size of 500–600 nm. Using XRD analysis, KNN thin films were found to have a perovskite structure without clear preferential orientation in the case of 5% potassium excess solution, whereas a texture in (100) direction appears for 20% alkali excess solutions, being particularly strong for the 0.4 M solution concentration. As a result of the grain size and (100) texturing competition, the highest room-temperature dielectric permittivity and lowest dissipation factor measured in the parallel-plate-capacitor geometry were obtained for KNN films deposited on platinized Al_2_O_3_ substrates using 0.2 M precursor solution with 20% alkali excess. Moreover, these films were shown to possess more quadratic-like and less coercive local piezoelectric loops, compared to those from 5% potassium excess solution. On the other hand, the highest piezoresponse was observed for KNN films prepared from 0.4 M precursor solution with 20% alkali excess that was attributed to a combination of both relatively large and nanoscale domain microstructures. Thus, it is shown that variation of ε_r_, tanδ, and piezoresponse of sol–gel derived KNN thin films can be achieved by controlling their grain size and texturing via the choice of the solution conditions.

## Figures and Tables

**Table 1:** Identification and properties of K_0.5_Na_0.5_NbO_3_ (KNN) thin films deposited on platinized Al_2_O_3_ substrates using three solution kinds.

Film Identifica-tion	K + Na Excess (%)	Molar Concen-tration	RMS Roughness (nm)	Average Grain Size (nm)	Film Thickness (nm)	(K+Na)/Nb	Lotgering Factor f_(100)_	ε_r_ @10 kHz	tan δ @10 kHz
5%_0.2 M	5 + 0	0.2	~39	~110	~340	1.01	0.13	110	0.364
20%_0.2 M	20 + 20	0.2	~38	~480	~330	1.08	0.39	177	0.094
20%_0.4 M	20 + 20	0.4	~35	~600	~350	1.07	0.72	103	0.224

## Supplementary Materials

The following are available online at https://www.mdpi.com/2079-4991/9/11/1600/s1, Figure S1: K, Na, Nb, Pt and Al elemental maps as well as cross-section micrograph of KNN thin films deposited on platinized Al_2_O_3_ substrates from solutions with 5% excess of potassium and 0.2 M concentration, Figure S2: K, Na, Nb, Pt and Al elemental maps as well as cross-section micrograph of KNN thin films deposited on platinized Al_2_O_3_ substrates from solutions with 20% excess of potassium and sodium and 0.2 M concentration, Figure S3: K, Na, Nb, Pt and Al elemental maps as well as cross-section micrograph of KNN thin films deposited on platinized Al_2_O_3_ substrates from solutions with 20% excess of potassium and sodium and 0.4 M concentration.
