# Supplementary material for: Effect of Solution Conditions on the Properties of Sol–Gel Derived Potassium Sodium Niobate Thin Films on Platinized Sapphire Substrates
Source: Nanomaterials (Basel). 2019 Nov 11;9(11):1600. doi: 10.3390/nano9111600 (PMC6915527; doi:10.3390/nano9111600)
Supplement: Supplementary file 1 [file nanomaterials-09-01600-s001.pdf]

Supplementary Materials:

Effect of Solution Conditions on the Properties of Sol–Gel Derived Potassium Sodium Niobate Thin Films on Platinized Sapphire Substrates

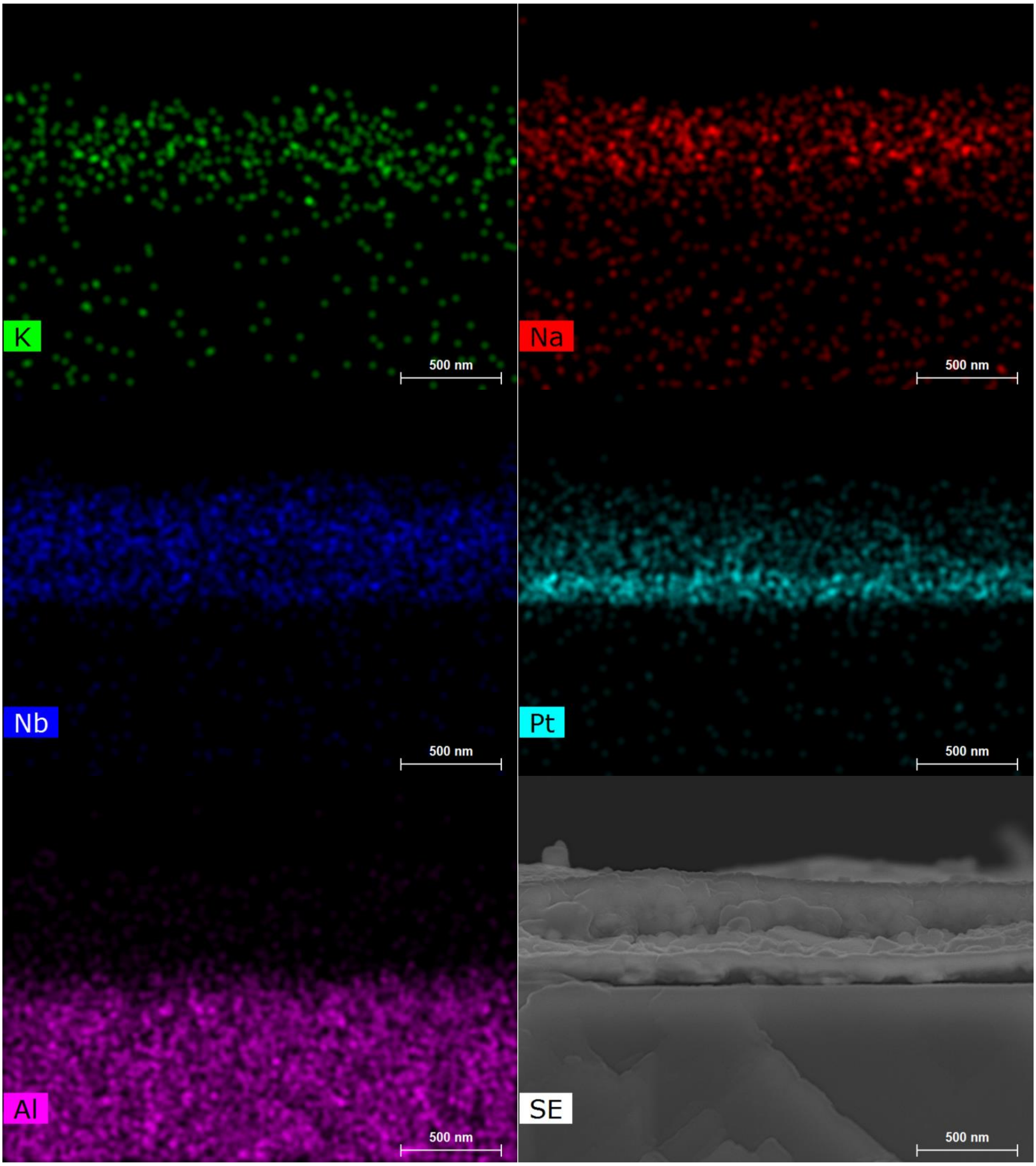

Figure S1. K, Na, Nb, Pt and Al elemental maps as well as cross-section micrograph of KNN thin films deposited on platinized Al_2O_3 substrates from solutions with 5% excess of potassium and 0.2 M concentration.

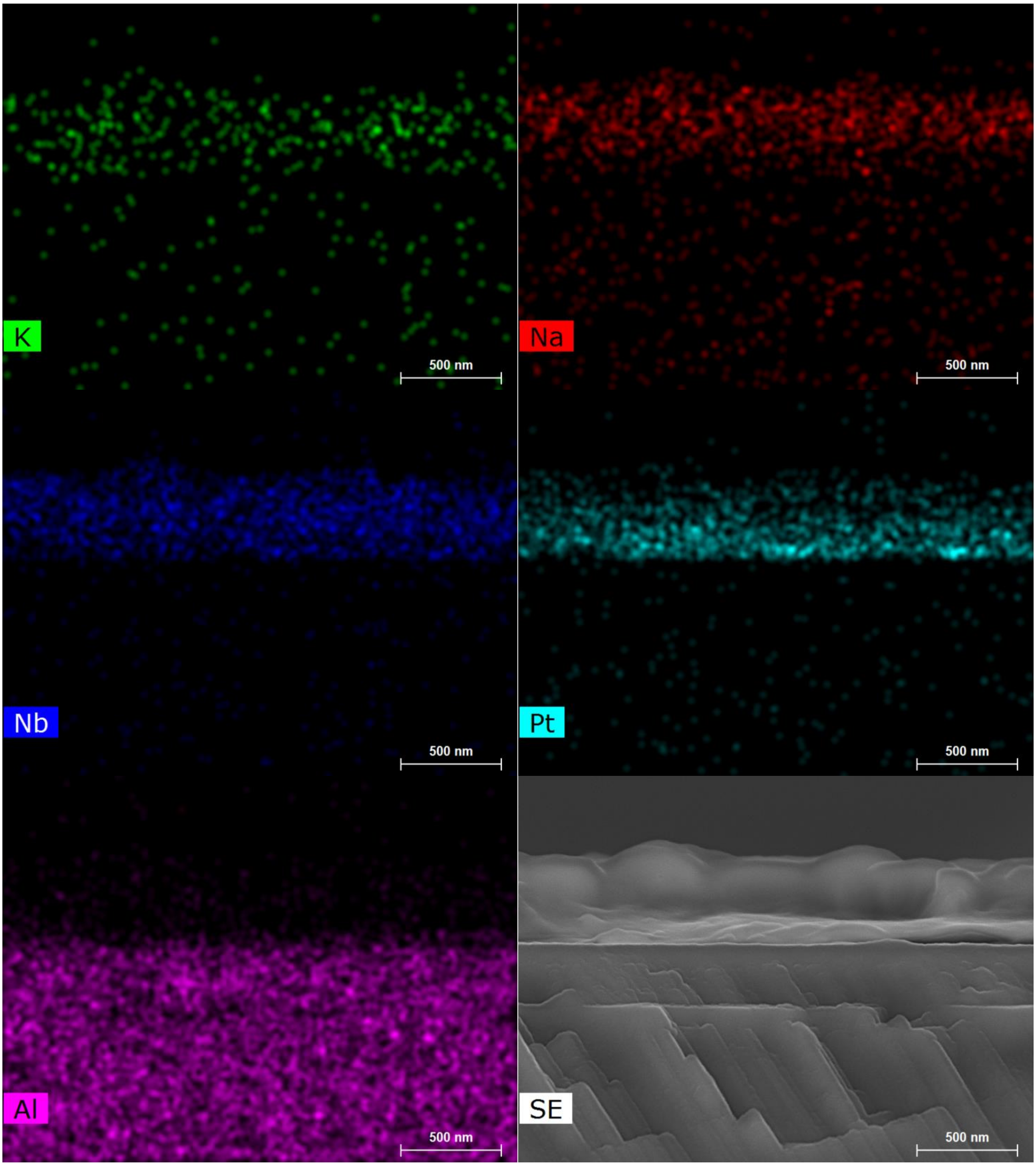

Figure S2. K, Na, Nb, Pt and Al elemental maps as well as cross-section micrograph of KNN thin films deposited on platinized Al_2O_3 substrates from solutions with 20% excess of potassium and sodium and 0.2 M concentration.

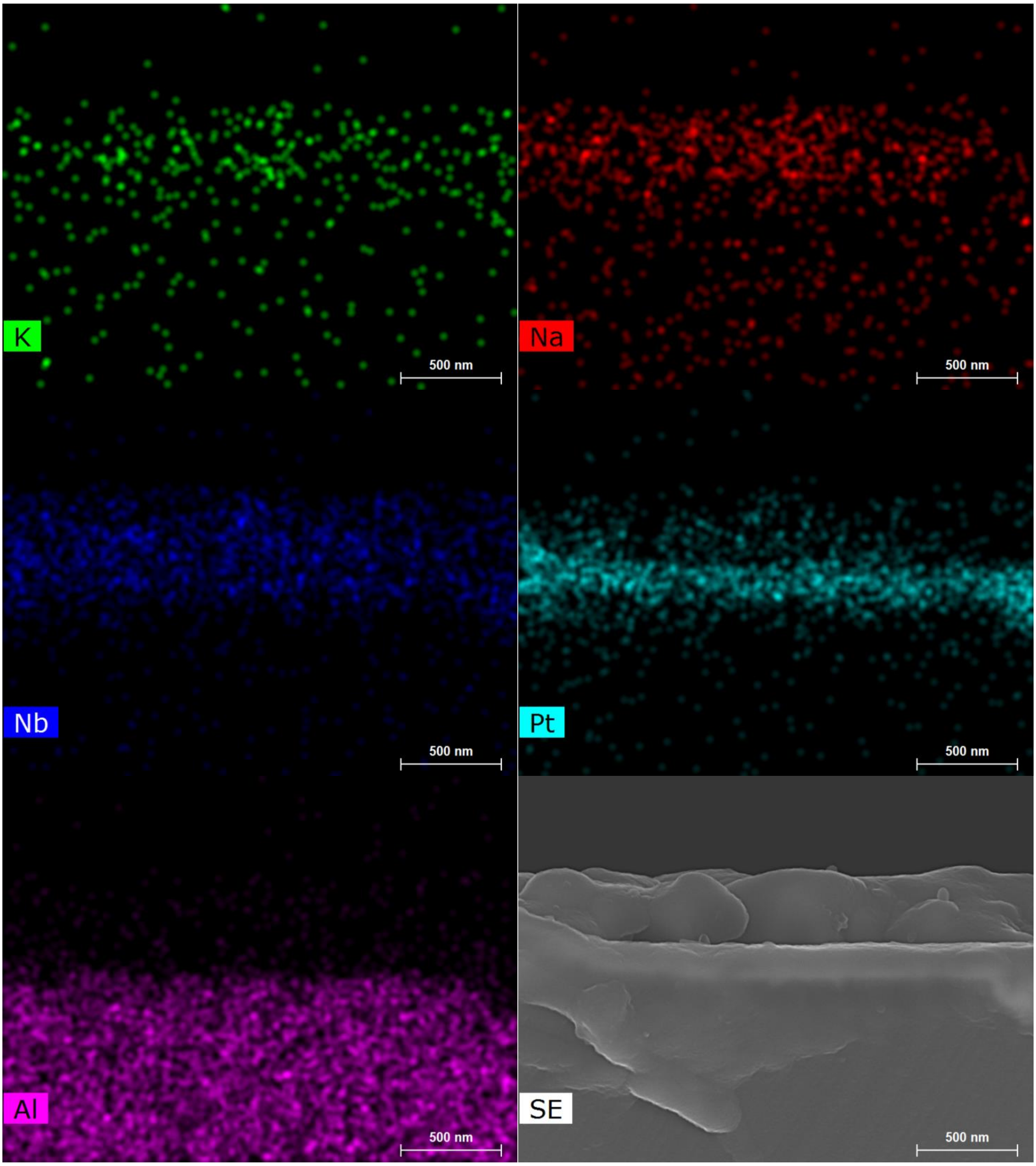

Figure S3. K, Na, Nb, Pt and Al elemental maps as well as cross-section micrograph of KNN thin films deposited on platinized Al_2O_3 substrates from solutions with 20% excess of potassium and sodium and 0.4 M concentration.
